# Housing First and harm reduction: a rapid review and document analysis of the US and Canadian open-access literature

**DOI:** 10.1186/s12954-017-0158-x

**Published:** 2017-05-23

**Authors:** Dennis P. Watson, Valery Shuman, James Kowalsky, Elizabeth Golembiewski, Molly Brown

**Affiliations:** 1 Department of Social and Behavioral Sciences, Indiana University Fairbanks School of Public Health, 1050 Wishard Blvd, Indianapolis, IN 46202 USA; 2 Heartland Health Outreach, Midwest Harm Reduction Institute, 1207 W. Leland Ave., Chicago, IL 60640 USA; 3 Department of Health Policy and Management, Indiana University Fairbanks School of Public Health, 1050 Wishard Blvd, Indianapolis, IN 46202 USA; 4 Department of Psychology, DePaul University, 2219 N. Kenmore Ave., Chicago, IL 60614 USA

**Keywords:** Harm reduction, Housing First, Rapid review, Document analysis, Housing, Substance use, Drug use, Implementation, Fidelity

## Abstract

**Background:**

Housing First is an evidence-based practice intended to serve chronically homeless individuals with co-occurring serious mental illness and substance use disorders. Despite housing active substance users, harm reduction is an often-overlooked element during the Housing First implementation process in real-world settings. In this paper, we explore the representation of the Housing First model within the open-access scholarly literature as a potential contributing factor for this oversight.

**Methods:**

We conducted a rapid review of the US and Canadian open-access Housing First literature. We followed a document analysis approach, to form an interpretation of the articles’ content related to our primary research questions.

**Results:**

A total of 55 articles on Housing First were included in the final analysis. Only 21 of these articles (38.1%) included explicit mention of harm reduction. Of the 34 articles that did not discuss harm reduction, 22 provided a description of the Housing First model indicating it does not require abstinence from substance use; however, descriptions did not all clearly indicate abstinence was not required beyond program entry. Additional Housing First descriptions focused on the low-barrier entry criteria and/or the intervention’s client-centeredness.

**Conclusions:**

Our review demonstrated a lack of both explicit mention and informed discussion of harm reduction in the Housing First literature, which is likely contributing to the Housing First research-practice gap to some degree. Future Housing First literature should accurately explain the role of harm reduction when discussing it in the context of Housing First programming, and public agencies promoting Housing First uptake should provide resources for proper implementation and monitor program fidelity to prevent model drift.

## Background

Developed in the 1990s [1], Housing First is an evidence-based practice that is the focal point of the US and Canadian governments’ approaches to addressing homelessness today [2, 3]. The model was developed to specifically serve chronically homeless individuals with co-occurring serious mental illness and substance use disorders. In contrast to more traditional housing approaches, Housing First does not require sobriety or treatment/service compliance as a condition for program entry or service continuation. As such, one of the key ingredients for a successful Housing First program is harm reduction-informed services [4–6]. In the context of the Housing First model, harm reduction is comprehensive and implemented with regard to substance use, management of psychiatric symptoms, and other areas of clients’ lives that might make them vulnerable to harm [7]. Within a Housing First program, providers should actively and assertively engage clients in harm reduction strategies and utilize motivational interviewing techniques to guide them toward achieving their stated recovery goals [8]. Despite its status as an essential component of the intervention, harm reduction is often overlooked during the Housing First implementation process in real-world settings [5, 9, 10]. For instance, Watson et al. [5] found 18 of the 39 Housing First programs in their national sample were operating without harm reduction policies and procedures despite the presence of other key elements of program fidelity. In this paper, we explore the representation of the Housing First model within the open-access academic literature as one potential reason contributing to this oversight. Before focusing specifically on this issue, we provide an overview of some of the factors that have impeded the implementation of harm reduction in Housing First programs.

### Factors impeding harm reduction implementation

Many problems related to the implementation of evidence-based practices can be traced to miscommunications or a lack of detailed explanation of interventions within the scientific literature [11, 12], and Housing First is no exception. Several misunderstandings of the Housing First model, including the essentialness of harm reduction, can be traced to the lack of fidelity guidelines at the time of its original diffusion across the USA in the early 2000s. Indeed, the US Department of Housing and Urban Development (HUD) commissioned a study more than 5 years after the initial push toward Housing First to begin to understand what fidelity to the model actually meant [13], and the first empirically based fidelity scales (which included harm reduction as a component) were not published until 2013 [5, 8]. Before fidelity guidelines were available, understandings of the model were formed as the result of a “telephone game” where housing providers obtained their information from documents produced by government and advocacy agencies with poor model descriptions that did not articulate elements of the harm reduction approach defined in later published fidelity scales [14, 15]. This resulted in many US housing organizations implementing low-barrier entry requirements allowing active substance users (and other individuals engaged in various risky behaviors) without the harm reduction strategies necessary to keep them housed [5]. Demonstrating how this looks on the ground, though their work providing training and technical assistance, the first three authors of this paper regularly encounter Housing First case managers who are housing active substance users but do not use or cannot define what harm reduction is.

Another factor negatively impacting implementation of the Housing First model is resistance to harm reduction. Although it continues today, harm reduction was a much more politically charged topic in the USA before the Obama administration [16]. Indeed, in their 2007 HUD report, Pearson and colleagues [15] explain in a footnote that they chose to use the term “low-demand” in place of “harm reduction” due to the contentious nature of the term at the time their study was conducted. This choice in itself may have led to further misinterpretation of Housing First by service providers: in social service practice, “low-demand” is a term often applied to lowered service requirements, rather than an active approach to working with individuals who use substances. Additionally, commitment to abstinence-only approaches is common among people who work with substance users [17, 18], and this commitment is a noted barrier to the implementation of harm reduction in Housing First programs [10].

Finally, there has been a move toward a system-wide Housing First approach in the USA, which understands Housing First to be a general philosophy, rather than a specified program model [19]. A Housing First system places emphasis squarely on the low-barrier admission criteria, without as much attention paid to the ways to work with people once they are housed. As such, Housing First has become conflated with other low-barrier housing approaches, such as rapid re-housing. One of the key tools of this approach is a coordinated entry system that utilizes a community-wide and centralized intake to match individuals with low-barrier housing offering varying levels of support and financial services based on a combination of their particular needs, program eligibility criteria, and unit availability [20]. As researchers and practitioners working in the area of Housing First implementation, we have observed confusion on the part of housing providers who believe every program in their community is a Housing First program because they use coordinated entry. The former Deputy Director of the US Interagency Council on Homelessness has advocated for thinking of Housing First as a systems approach, arguing its framing as a program creates “a dynamic in which individual programs are pitted against one another” ([21], par. 7). However, this statement overlooks the fundamental fact that *Housing First programming is evidence-based*, while programs within a Housing First system operating using different program models are not.

The view of homelessness solutions in the USA is starting to change as more advocacy and government organizations are starting to place emphasis on harm reduction as a key component of the model (see [22]), which is likely owed to the development and publication of the fidelity guidelines previously discussed. However, based on our experience conducting a current Housing First implementation study [10] and additional work delivering Housing First technical assistance and training, significant barriers to the implementation of harm reduction still exist. In this paper, we are particularly interested in understanding the extent to which the open-access scientific literature may be contributing to this issue. As such, we conducted a rapid review to understand how harm reduction was discussed within the available open-access Housing First literature in the USA and Canada.

Our reason for widening the focus beyond the USA is because Canada recently placed Housing First at the center of its housing strategy after demonstrated success of a multi-city randomized Housing First trial that followed strict fidelity criteria [2]. Furthermore, significant efforts to disseminate research among policymakers and service providers have been made in Canada. For example, the Canadian Observatory on Homelessness has developed the Homeless Hub [23], a comprehensive repository of homelessness and Housing First literature designed for dissemination among researchers, policymakers, and service providers. We chose to focus on open-access literature for several reasons. First, paywall barriers make it unlikely that non-open-access articles are heavily accessed by, and thus significantly influencing, the housing practice community, which primarily works for non-profit organizations with constrained resources (a fact well known by the second and third authors of this study who both work in this arena and regularly interact with those seeking Housing First resources as technical assistance and training providers). Second, websites, such as the Homeless Hub, may be “go-to” resources for service and training providers. However, even research dissemination sites such as this only provide open-access articles in full text due to journal subscription costs. Third, it has been hypothesized that because open-access articles are generally cited more rapidly and more often than non-open-access articles, knowledge translation to the broader community is also more likely for open-access research due to its accessibility [24, 25]. Finally, it has been argued that the availability of open-access literature to practitioners is a critical component of evidence-based practice in community agencies [26]. Thus, the primary questions guiding this review were as follows: (1) To what degree does the open-access Housing First literature discuss harm reduction?; (2) How is harm reduction discussed in this literature?; and (3) How do articles that do not discuss harm reduction describe the Housing First model?

## Methods

Rapid reviews are appropriate in cases where there is a need to develop a relatively comprehensive understanding of a well-defined issue within a short time frame and are generally conducted between 1 and 6 months [27–29]. To meet the deadline for the special issue of this journal, we conducted our review between December 3, 2016, and February 24, 2017. The stages in our review process included (1) problem formulation, (2) defining inclusion and exclusion criteria for the search, (3) identification and screening of the literature, and (4) data analysis. We have already outlined the formulation of the problem this study seeks to address in the last paragraph of the previous section.

### Defined parameters

To be included in our review, articles had to (1) focus on housing programming for homeless individuals; (2) discuss a Housing First program or general Housing First practice in the USA or Canada (simply referencing Housing First was not enough to meet inclusion); (3) be empirical (e.g., quantitative, qualitative, or mixed-method research, or a systematic review), non-empirical (e.g., theoretical, opinion-based and letter to the editor), or a study protocol (as protocols often have strong descriptions of interventions being tested); and (4) the article must be written in English.

### Literature identification and screening

**Fig. 1:**
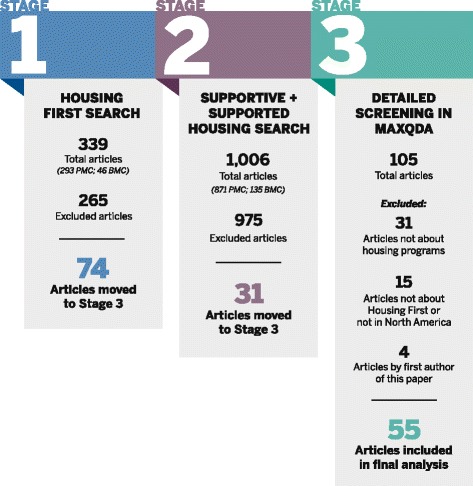
Description of article identification and inclusion/exemption for all review stages

Our literature screening process comprised three steps (see Fig. 1). In step 1, we searched PubMed Central (PMC) and BioMed Central (BMC) for full-text articles containing the phrase “Housing First” anywhere within them. This search yielded 339 total articles (including duplicates), which were all pulled into Zotero bibliographic management software [30]. We then removed all duplicate articles and articles with titles clearly indicating they were not about Housing First programming, and we searched article abstracts when the title was not clear, which left us with 74 unique articles that were moved forward for more detailed screening.

In step 2, we searched the same databases for the phrases “supportive housing” and “supported housing” located anywhere within an article (our logic for this search being some programs that follow a Housing First approach might not have been described as such within their respective article). This search identified 871 total articles (including duplicates), which we pulled into Zotero. We applied the same title and abstract screening criteria as well as removing duplicates of articles found in step 1, which left 31 that were moved on for more detailed screening.

In step 3, we conducted more detailed screening of the 105 unique articles identified in the previous steps to locate and remove those not fitting inclusion criteria. We first imported all articles that made it past the first two steps into MAXQDA qualitative data analysis software [31]. Next, we focused specifically on the articles obtained in step 2 since, not having the words “Housing First” in them, they were most likely not to fit inclusion criteria. We identified and removed all step 2 articles that did not discuss programs targeting the homeless population, did not focus specifically on housing programming, and articles that discussed the homeless population in general. We did this by first using MAXQDA’s query function to search articles not containing the string “homeless” anywhere within them and then reading the abstracts of all articles that did. At the end of this process, no unique articles identified in step 2 were demonstrated to meet our inclusion. We then thoroughly read all articles left to identify whether they were about Housing First programming in the USA or Canada and removed those that were not. We also removed all articles published by the authors of this paper, as their inclusion in the data would constitute circular logic since their work helped frame the problem that is the basis of this project. Step 3 screening resulted in a total of 55 articles that were moved to the analysis stage.

### Data analysis

Unlike most systematic reviews, the goal of our analysis was not to evaluate the quality and results of identified literature or to integrate findings into coherent themes. Rather, we aimed to understand the representation of particular issues (e.g., descriptions of harm reduction and the Housing First model) regardless of a paper’s results. As such, we conducted a document analysis, focusing on the articles’ content related to the primary questions guiding the study to form interpretation of the textual data [32–34].

The first author carried out the analysis using MAXQDA. All articles were identified before any analysis began. As a first step, he categorized all 55 articles into one of the following two sets: (1) Housing First articles discussing harm reduction (HFwithHR) or (2) Housing First articles that did not discuss harm reduction (HFnoHR). To accomplish this, he first used the lexical search function in MAXQDA to identify all articles with the string “harm reduction” in them, placing them in the HFwithHR set, and moved all other articles into the HFnoHR set. Then he examined the placements of “harm reduction” in the articles and moved all articles where the string appeared only in the reference section to the HFnoHR set. Analysis of each document set diverged at this point due to the research questions.

As a first step in this analysis, the first author reviewed the articles and developed an initial coding scheme based on the content with separate sets of codes for each document set. For the HFwithHR set, codes were developed inductively by looking at the sections of the articles where Housing First was discussed and paying attention to how harm reduction was described in relation to the Housing First model. For the HFnoHR set, we were mainly interested in how they described the Housing First model without discussing harm reduction. We were particularly interested in whether the articles described sobriety requirements as being in opposition to the Housing First model and, if so, to what degree. Therefore, this set was coded deductively by creating three categories to sort the articles into based on how they explained the Housing First model: (1) Housing First not sufficiently explained, (2) Housing First described as not having sobriety requirements, and (3) Housing First described as being low-barrier, flexible, or emphasizing choice without explicitly discussing how sobriety requirements relate to the model. During analysis of the HFnoHR articles, the primary description of the Housing First model within the article was focused on, rather than isolated comments regarding it.

While all coding was conducted by the first author, we selected six articles (10% of the sample) to be coded again by the fourth author to check for interrater agreement as a means of assessing the clarity of code definitions (the second coder was EG). We obtained 78% agreement (kappa = 0.74), indicating a moderate level of agreement, which is more than appropriate for an interpretive analysis [35].

## Results

### Sample description

Table 1 displays basic information regarding articles in the sample (*n* = 55). The years of publication ranged from 2004 to 2016, and the year with the largest number of publications (*n* = 11) was 2013. Forty-eight publications were research articles, three were protocols, three were editorials or opinions, and one was a literature review. Thirty-seven were US articles and 18 were Canadian. Thirty-eight articles mentioned harm reduction somewhere in them, but only 21 mentioned it in the main text (i.e., parts of the article not including references or subtitles), resulting in 21 HFwithHR articles and 34 HFnoHR articles. Findings related to the analyses for each article set are presented separately below. Page numbers for quoted material reflect the location in the open-access versions of the articles.

**Table 1:** Articles included in the literature review arranged by year of publication

Author(s)	Year	Title	Article type	Country	Harm reduction mentioned
Tsemberis, Gulcur, and Nakae [70]	2004	Housing First, consumer choice, and harm reduction for homeless individuals with a dual diagnosis	Research	USA	Yes
Milby, Schumacher, Wallace, Freedman, and Vuchinich [71]	2005	To house or not to house: the effects of providing housing to homeless substance abusers in treatment	Research	USA	No
Dickson-Gomez, Convey, Hilario, Corbett, and Weeks [72]	2007	Unofficial policy: access to housing, housing information and social services among homeless drug users in Hartford, Connecticut	Research	USA	No
Kertesz et al. [59]	2007	Long-term housing and work outcomes among treated cocaine-dependent homeless persons	Research	USA	No
Padgett [38]	2007	There’s no place like (a) home: ontological security among persons with serious mental illness in the United States	Research	USA	Yes
Buchanan, Kee, Sadowski, and Garcia [56]	2009	The health impact of supportive housing for HIV-positive homeless patients: a randomized controlled trial	Research	USA	No
Kertesz, Crouch, Milby, Cusimano, and Schumacher [44]	2009	Housing First for homeless persons with active addiction: are we overreaching?	Research	USA	Yes
Parker [58]	2010	Housing as an intervention on hospital use: access among chronically homeless persons with disabilities	Research	USA	No
Tsai, Mares, and Rosenheck [57]	2010	A multisite comparison of supported housing for chronically homeless adults: “Housing First” versus “residential treatment first”	Research	USA	No
Fitzpatrick-Lewis et al. [47]	2011	Effectiveness of interventions to improve the health and housing status of homeless people: a rapid systematic review	Literature review	USA	No
Goering et al. [53]	2011	The At Home/Chez Soi trial protocol: a pragmatic, multi-site, randomised controlled trial of a Housing First intervention for homeless individuals with mental illness in five Canadian cities	Protocol	Canada	No
Henwood, Stanhope, and Padgett [50]	2011	The role of housing: a comparison of front-line provider views in housing first and traditional programs	Research	USA	No
Raven, Doran, Kostrowski, Gillespie, and Elbel [73]	2011	An intervention to improve care and reduce costs for high-risk patients with frequent hospital admissions: a pilot study	Research	USA	Yes
Padgett, Stanhope, Henwood, and Stefancic [74]	2011	Substance use outcomes among homeless clients with serious mental illness: comparing Housing First with treatment first programs	Research	USA	Yes
Collins, Malone, Clifasefi, et al. [75]	2012	Project-based Housing First for chronically homeless individuals with alcohol problems: within-subjects analyses of 2-year alcohol trajectories	Research	USA	No
Hwang, Stergiopoulos, O’Campo, and Gozdzik [76]	2012	Ending homelessness among people with mental illness: the At Home/Chez Soi randomized trial of a Housing First intervention in Toronto	Research	Canada	Yes
Tsemberis, Kent, and Respress [77]	2012	Housing stability and recovery among chronically homeless persons with co-occuring disorders in Washington, DC	Research	USA	Yes
Stergiopoulos et al. [78]	2012	Moving from rhetoric to reality: adapting Housing First for homeless individuals with mental illness from ethno-racial groups	Research	Canada	Yes
Collins, Clifasefi, Andrasik, et al. [45]	2012	Exploring transitions within a project-based Housing First setting: qualitative evaluation and practice implications	Research	USA	Yes
Collins, Malone, and Larimer [41]	2012	Motivation to change and treatment attendance as predictors of alcohol-use outcomes among project-based Housing First residents	Research	USA	Yes
Collins, Clifasefi, Dana, et al. [63]	2012	Where harm reduction meets housing first: exploring alcohol’s role in a project-based Housing First setting	Research	USA	Yes
Collins, Malone, and Clifasefi [79]	2013	Housing retention in single-site Housing First for chronically homeless individuals with severe alcohol problems	Research	USA	No
Henwood, Shinn, Tsemberis, and Padgett [40]	2013	Examining provider perspectives within Housing First and traditional programs	Research	USA	Yes
Henwood, Stanhope, et al. [62]	2013	Addressing chronic disease within supportive housing programs	Research	USA	No
O’Toole et al. [60]	2013	New to care: demands on a health system when homeless veterans are enrolled in a medical home model	Research	USA	No
Owczarzak, Dickson-Gomez, Convey, and Weeks, [36]	2013	What is “support” in supportive housing: client and service providers’ perspectives	Research	USA	Yes
Palepu, Patterson, Moniruzzaman, Frankish, and Somers [48]	2013	Housing First improves residential stability in homeless adults with concurrent substance dependence and mental disorders	Research	Canada	No
Patterson, Rezansoff, Currie, and Somers [54]	2013	Trajectories of recovery among homeless adults with mental illness who participated in a randomised controlled trial of Housing First: a longitudinal, narrative analysis	Research	Canada	No
Somers, Patterson, et al. [61]	2013	Vancouver At Home: pragmatic randomized trials investigating Housing First for homeless and mentally ill adults	Research	Canada	No
Somers, Rezansoff, Moniruzzaman, Palepu, and Patterson [42]	2013	Housing First reduces re-offending among formerly homeless adults with mental disorders: results of a randomized controlled trial	Research	Canada	Yes
Srebnik, Connor, and Sylla [80]	2013	A pilot study of the impact of Housing First–supported housing for intensive users of medical hospitalization and sobering services	Research	USA	Yes
Stefancic et al. [37]	2013	Implementing Housing First in rural areas: Pathways Vermont	Research	USA	Yes
Adair et al. [55]	2014	Development and initial validation of the Observer-Rated Housing Quality Scale (OHQS) in a multisite trial of Housing First	Research	Canada	No
Farquhar, Ryder, Henderlong, Lowe, and Amann [81]	2014	Listening to consumer perspectives to inform addictions and housing-related practice and research	Research	USA	No
Fleury, Grenier, and Vallée [82]	2014	Evaluation of the implementation of the Montreal At Home/Chez Soi project	Research	Canada	No
Kertesz et al. [83]	2014	Making Housing First happen: organizational leadership in VA’s expansion of permanent supportive housing	Research	USA	Yes
Kirst, Zerger, Harris, Plenert, and Stergiopoulos [52]	2014	The promise of recovery: narratives of hope among homeless individuals with mental illness participating in a Housing First randomised controlled trial in Toronto, Canada	Research	Canada	No
Mackelprang, Collins, and Clifasefi [84]	2014	Housing First is associated with reduced use of emergency medical services	Research	USA	No
Stergiopoulos et al. [39]	2014	Housing First: exploring participants’ early support needs	Research	Canada	Yes
Aubry, Nelson, and Tsemberis [85]	2015	Housing First for people with severe mental illness who are homeless: a review of the research and findings from the At Home–Chez Soi demonstration project	Research	Canada	No
Goering and Streiner [86]	2015	Putting housing first: the evidence and impact	Editorial/opinion	Canada	No
Henwood, Byrne, and Scriber [87]	2015	Examining mortality among formerly homeless adults enrolled in Housing First: an observational study	Research	USA	No
Henwood, Stefancic, et al. [88]	2015	Social relationships of dually diagnosed homeless adults following enrollment in Housing First or traditional treatment services	Research	USA	No
Kertesz, Austin, Holmes, Pollio, and Lukas [89]	2015	Housing First and the risk of failure: a comment on Westermeyer and Lee (2013)	Editorial/opinion	USA	Yes
Ly and Latimer [90]	2015	Housing First impact on costs and associated cost offsets: a review of the literature	Research	USA	No
Smelson [91]	2015	A cluster randomized Hybrid Type III trial testing an implementation support strategy to facilitate the use of an evidence-based practice in VA homeless programs	Protocol	USA	No
Stergiopoulos et al. [43]	2015	Effectiveness of Housing First with intensive case management in an ethnically diverse sample of homeless adults with mental illness: a randomized controlled trial	Research	Canada	Yes
Woodhall-Melnik et al. [49]	2015	The impact of a 24-month Housing First intervention on participants’ body mass index and waist circumference: Results from the At Home/Chez Soi Toronto site randomized controlled trial	Research	Canada	No
Brown et al. [92]	2016	Pathways to homelessness among older homeless adults: results from the HOPE HOME study	Research	USA	No
Gabrielian et al. [93]	2016	Factors associated with premature exits from supported housing	Research	USA	No
Kennedy et al. [94]	2016	A computer-assisted motivational social network intervention to reduce alcohol, drug and HIV risk behaviors among Housing First residents	Protocol	USA	No
Killaspy [95]	2016	Supported accommodation for people with mental health problems	Editorial/opinion	USA	No
O’Campo et al. [96]	2016	How did a Housing First intervention improve health and social outcomes among homeless adults with mental illness in Toronto? Two-year outcomes from a randomised trial	Research	Canada	No
Stergiopoulos et al. [97]	2016	The effectiveness of a Housing First adaptation for ethnic minority groups: findings of a pragmatic randomized controlled trial	Research	Canada	No
Pauly et al. [46]	2016	Finding safety: a pilot study of managed alcohol program participants’ perceptions of housing and quality of life	Research	Canada	Yes

### How harm reduction was discussed in articles mentioning harm reduction

Of the 21 HFwithHR articles, the number of times harm reduction was mentioned in the main text ranged between 1 and 28 times with an average of 4.2 (SD = 6.2) mentions. Only the article by Owczarzak et al. [36] did not discuss the relationship between harm reduction and Housing First in some way. Rather, they discussed it as a treatment approach used by some supportive housing programs.

Fifteen of the HFwithHR articles explicitly stated harm reduction was part of the Housing First model or used wording strongly suggesting the two approaches are part of a package deal; however, one only made this statement parenthetically [37]. Padgett [38] clearly discusses Pathways to Housing as the original Housing First program that “departed from the ‘treatment first’ approach by offering [among a list of other components mentioned] harm reduction with respect to mental health treatment and substance abuse….” (p. 4–5). Stergiopoulos et al. [39] clearly demonstrate the connection between Housing First and harm reduction when they state “a harm reduction approach is followed” (p. 2) in Housing First programs. Though less strongly worded, Henwood et al. [40] discuss how Housing First “openly embraces harm reduction” (p. 2) and how harm reduction has been adopted as a “general framework” (p. 5) by the Housing First model.

Five articles discussed harm reduction as being compatible with Housing First practice or simply present in a program(s) without explicitly stating it was part of the model. The following statement by Collins et al. [41] provides an example of this: “Housing First is therefore consistent with harm reduction approaches, which deemphasize pathologizing alcohol use and support the realization of client-driven goals that can reduce harm and improve quality of life” (p. 2). In the case of an article by Somers et al. [42], the authors simply point out that “a harm reduction approach to substance use was promoted” (p. 3) in the program they studied. Similarly, Stergiopoulos et al. [43] state Housing First “service teams offered [intensive case management] using a recovery oriented, trauma informed approach and harm reduction principles” (p. 4) in the program they studied.

Ten articles actually described what harm reduction encompasses. Of these, only eight were also articles that had described harm reduction as a component of or consistent with the Housing First model. Seven articles described harm reduction only as it relates to substance use. These definitions ranged all the way from simply stating harm reduction was not concerned with abstinence from substance use [44] to more nuanced explanations such as Collins et al. [45] who state harm reduction uses:"…pragmatic strategies to minimize substance-related, negative consequences, while maintaining a nonjudgmental, empathetic stance and supporting the realization of client-driven versus provider-driven goals" (p. 2). While not drawing the connection between harm reduction and Housing First, Owczarzak et al. [36] emphasized the various life areas—“social, legal, economic, and biological” (p. 3)—that harm reduction seeks to reduce the related negative consequences for. Pauly et al. [46] went beyond the individual client, discussing how harm reduction can focus on “safer settings (physical environments), organizational and governmental policies and practices that shift social, economic, and policy environments” (p. 3).

Only two articles used language suggesting harm reduction could focus on more than just substance use. In their results, Henwood et al. [40] used a quote from a Housing First provider demonstrating how harm reduction is about more than just substance use in that “It extends not just with drug use. It expands with working and a whole slew of things, relationships, and you can apply it to almost every service you provide” (p. 5). Somers et al. [42] stated that harm reduction was applied generally by staff toward risky behaviors; however, they parenthetically offer non-abstinence-based substance use treatment as their only example.

Of the ten articles that described harm reduction, five also contrasted it directly with traditional continuum of care or abstinence-only approaches to housing service provision. For example, Collins et al. [45] stated:

One of the fundamental theoretical differences between the continuum/medical and Housing First/harm reduction models lies in the understanding of the mechanism by which individuals are likely to change their behavior. The continuum/medical model holds that alcohol behavior change…is optimally achieved through…treatment attendance and rewarding more “desirable” behavior…In contrast, the Housing First/harm reduction model is built on the assertion that behavior change is most lasting if it is client-driven….(p. 2)

The above selection from Collins et al. was the most detailed example, as the other articles simply pointed to the models as “different” or contrasted how they approach substance use without delving into the specific logics underlying them. However, Pauly et al. [46] provided a unique contrast in that they pointed to the controversial nature of harm reduction because it was aimed “to reduce the harms of substance use rather than promote abstinence or reduce substance use” (p. 9), where other articles simply stated the continuum of care/abstinence-only approach was more ubiquitous.

### Discussions of Housing First in articles not mentioning harm reduction

Of the 34 HFnoHR articles, only one, a literature review of various housing interventions by Fitzpatrick [47], did not provide any description of the Housing First model. Twenty-two of these articles described Housing First in a manner indicating it “does not require abstinence from drugs and alcohol among clients” [48] (p. e34). The following selection from Woodhall-Melnik et al. [49] offers one of the strongest descriptions of the way Housing First programs approach substance use, which would be consistent with a harm reduction approach:

Housing First involves providing low-barrier, rapid access to housing and mental health support services wherein individuals are given access to independent housing with no sobriety or mental health treatment enrollment or compliance requirements. (p. 2)

The strength of this description lies in its discussion of both criteria for program eligibility and continuance of housing as not requiring abstinence. Articles by Henwood et al. [50] and Fleury et al. [51] provided similarly strong examples.

In contrast to this, 13 articles only stated sobriety was not an eligibility requirement for housing access without discussing how Housing First programs dealt with substance use after clients were housed. For instance, Kirst et al. [52] state:

Housing First is an intervention for individuals experiencing homelessness and mental illness which places individuals into permanent independent Housing First—without prerequisites for sobriety and treatment—and offers flexible access to supportive health services. (p. 2)

"Immediate provision/access" was a common phrase used in conjunction with descriptions of Housing First’s lack of sobriety requirements for intake [37, 53–55].

Finally, there were four examples where the relationship between Housing First and substance use was not clearly stated or was convoluted. In three of these examples, Housing First’s rules around sobriety were described in contrast to traditional, abstinence-focused programming. Buchanan et al. [56] indicated:

Housing First is a theory that homeless individuals are best stabilized through housing regardless of the personal challenges they may experience. This contrasts with the traditional housing readiness system that preferentially houses more stable and organized individuals by requiring repeated follow-up visits, stable contact information, and often sobriety…. (p. S679)

In the article by Tsai et al. [57], the authors make the following statement that could be interpreted in a manner contrasting the Housing First model:

The Housing First program, as its name suggests, offers homeless clients immediate independent housing off the streets and attempts to find housing that satisfies their needs and preferences with only limited requirements for psychiatric treatment or sobriety. (p. 2)

The authors’ statement that Housing First offers “limited requirements…for sobriety” could be construed by some to mean sobriety requirements are compatible with the model on some level.

Ten articles did not discuss substance use as it relates to the Housing First model. And seven of these ten discussed Housing First as being “low-barrier” or providing “immediate access” to clients coming directly off the street, while also highlighting the importance of supportive services: “The Housing First methodology is a reversal of common practice in the United States by providing more immediate housing prior to supportive services.” ([58] p. 913). The articles by Kertesz et al. [59] and O’Toole et al. [60] simply mention the Housing First as being low-demand, meaning they did not require participation in any specific services.

Finally, five of the ten articles that did not directly discuss how Housing First approaches substance use, including three that had also discussed the model as “low-barrier,” described Housing First in terms of its client-centeredness. For instance, Somers et al. [61] focused primarily on the choice the model gives to clients regarding housing location:

Housing First emphasizes the value of client choice…Housing First involves building a portfolio of rental accommodations (typically apartments) scattered throughout different neighborhoods, thereby providing clients with meaningful choices concerning the location and setting of the residence. (p. 2)

Providing another example, Henwood at al. [62] discussed this issue more in terms of the tailoring of supportive services to the individual client:

[Housing First] programs provide immediate access to publicly subsidized housing rented from private landlords along with flexible supports designed specifically for individuals…the intensity of services is based on individual need. (p. 2)

## Discussion

Our findings demonstrate there is considerable variation in the extent to which harm reduction is described within a sample of US and Canadian open-access Housing First literature. And, while we cannot state there is a direct correlation between weak intervention portrayals and problems with Housing First implementation, our analysis does demonstrate that the inconsistent use of harm reduction demonstrated to exist in practice [5, 9, 10] is mirrored by inconsistent descriptions in the literature.

The majority of the articles in our sample did not discuss harm reduction within their main text, and there was significant variation among those that did regarding how harm reduction was discussed. While the majority of the HFwithHR articles explicitly identified harm reduction as part of Housing First, language discussing harm reduction as compatible with, rather than critical to, Housing First could be taken to mean harm reduction is an optional component of the intervention. More than half the HFwithHR articles did not clearly define harm reduction or how it is applied in Housing First programming. Finally, few HFwithHR described specific engagement strategies, such as motivational interviewing, that were used within the model to promote harm reduction. Therefore, housing providers may come away with a narrow view of the harm reduction approach as it is implemented in Housing First.

The emphasis placed on harm reduction practices appeared to be particularly common in articles focusing on populations with primary substance use disorders. For example, the HFwithHR article by Pauly and colleagues [46] and three HFwithHR articles by Collins and colleagues [41, 45, 63] described Housing First programs designed specifically for individuals with severe alcohol problems. A lack of emphasis on harm reduction in the broader Housing First literature runs the risk of conveying it is not a key element of the intervention unless substance use is the primary program focus. Further, it is possible housing providers, viewing specialty programs as unrelated to the services they provide, might not extend harm reduction approaches to their programs.

The fact that 18 of the HFnoHR articles referenced literature with “harm reduction” in their titles and HFnoHR authors also had articles appearing in the HFwithHR set indicates there is likely an awareness of the importance of harm reduction that is not evidenced in the main text of many publications. One likely reason for this is lack of significant space to include rich descriptive information due to scholarly journal page limitations [64]. One positive note is that the majority of the HFnoHR articles at least stated the model does not require abstinence of residents; however, many of these relayed information in such a way as to suggest abstinence was not a requirement of entry without clearly stating clients should not lose their housing due to substance use. Lack of clarity regarding this issue could account for why some programs feel as though they can require abstinence after program entry.

We realize guidance regarding program implementation is not the goal of most scholarly articles, and we do not expect researchers will begin providing detailed guidance on Housing First implementation any time soon. However, our findings do stress the importance for researchers to more accurately convey the role of harm reduction and the ways it is utilized when it is described in Housing First research and to consider moving beyond journal publication as a singular form of dissemination to help clear up misconceptions, improve fidelity of implementation, and close the research-practice gap [65, 66]. When doing this, we suggest authors focus on the definition of harm reduction provided in existing fidelity measures, which are highly compatible: “Participants are not required to abstain from alcohol and/or drugs and staff work consistently with participants to reduce the negative consequences of use…” ([67], p. 247); “Reduce the negative consequences related to substance abuse (and other high-risk behaviors) rather than eliminating substance use altogether” ([5], p. 16). In addition, drawing on texts with more comprehensive descriptions of the Housing First model (e.g., [7]) may provide a broader view of harm reduction elements in Housing First, such as its applicability to psychiatric symptoms and other harmful behaviors.

It is possible that the research-practice gap will widen in the USA due to the current move to a housing systems approach informed by a Housing First philosophy [19, 21]. This is because more programs existing within coordinated entry systems are likely to call themselves “Housing First” when they have not appropriately implemented the model. This could also lead to more negative perceptions of Housing First as programs that believe they are working within its parameters obtain negative service outcomes [68]. Perhaps a better term for these systems would be “low-barrier” or “coordinated entry” systems to avoid likely confusion. Another approach might be to require programs to demonstrate fidelity to the model if they wish to receive federal funding. While these issues are largely set within the US context, the lack of explicit discussion of harm reduction in more than 50% of the Canadian articles might foreshadow a drift in Housing First fidelity [69] as the model begins to disseminate in light of new government policies.

This project faces limitations common to any rapid review, namely we had to make precise decisions regarding our research questions and boundaries of the literature we focused on due to time constraints. While a look at the broader Housing First literature may have yielded a sample with richer data, focusing on the scholarly literature most available to policymakers and practitioners was the best for understanding how it may be contributing to the Housing First research-practice gap. Our findings did demonstrate that a broader systematic review of the Housing First literature, including the gray literature, would be a worthwhile endeavor to produce a stronger understanding of the representation of harm reduction within it, as well as how these messages are being interpreted and disseminated by government and professional organizations. To better understand issues affecting the translation of the Housing First model, future work could seek to better identify the extent to which the scientific literature and other possible factors (e.g., other pathways of information diffusion, culture, funding, politics) might be contributing to misinterpretations of the model through such methods as key stakeholder interviews or a survey of Housing First practitioners. This work would benefit work related to Housing First specifically, as well as the broader literature on program implementation.

## Conclusions

The findings of this review demonstrate a lack of both explicit mention and informed discussion of harm reduction in the scholarly open-access Housing First literature from the USA and Canada, which confirms assumptions based on previous literature and our experiences as Housing First researchers and professionals assisting with Housing First training and technical assistance. Future authors of scholarly literature should be weary to accurately explain the role of harm reduction when it is discussed in the context of Housing First programming. They should also explicitly refer readers to fidelity guidelines to avoid future problems with the implementation of harm reduction in real-world programs. Additionally, public and government agencies wishing to promote the evidence-based Housing First model should include guidance regarding essential program elements based on established fidelity guidelines, provide linkage to resources for programs and practitioners to build the necessary skills for success, and consider requiring fidelity assessments for programs labeling themselves “Housing First” in order to prevent excessive model drift.

## Abbreviations

HFnoHR: Articles in our sample without the string “harm reduction” in the main body of the text
HFwithHR: Articles in our sample with the string “harm reduction” in the main body of the text
HUD: US Department of Housing and Urban Development
